# Lucidity: Differences in Perspective

**DOI:** 10.7759/cureus.113085

**Published:** 2026-07-21

**Authors:** Sarah Joy Gallardo, Eli Rudnisky, Robert J Coni

**Affiliations:** 1 Office of Research and Sponsored Programs, Burrell College of Osteopathic Medicine, Melbourne, USA; 2 Department of Biomedical Sciences, Burrell College of Osteopathic Medicine, Melbourne, USA; 3 Department of Preclinical Medicine, Burrell College of Osteopathic Medicine, Melbourne, USA

**Keywords:** caregiver awareness, cognitive impairment and dementia, end-of-life and hospice care, paradoxical lucidity, physician awareness, terminal lucidity

## Abstract

The phenomenon of lucidity refers to transient episodes of cognitive clarity in individuals with advanced and irreversible neurological impairment, typically coupled with late-stage illness. Although there have been strides spanning the course of decades to understand what a lucid episode (LE) truly entails, research regarding this topic remains in its infantile stages. Using a hybrid narrative-systematic-based literature approach, this review aims to review the existing body of literature and provide between-group qualitative comparisons of LEs from family members and healthcare workers to identify shared characteristics, observer-related differences, and potential predictors of lucidity. A variety of sources were vetted for inclusion, including interviews, surveys, case reports, and observational analyses in patients with severe neurological diseases. Between-group similarities included the characterization and duration of LEs, while notable differences included potential triggers and the emotional appraisal of these episodes. Limitations of the study, such as heterogenous definitions, retrospective reporting, various forms of bias, inconsistent assessment tools, and the absence of neurobiological measures, appear to be reflective of the early stages of research regarding lucidity. Therefore, future research should prioritize conceptual refinement through a standardized definition and exclusion criteria, prospective study designs, and the integration of physiological and neurological monitoring in this patient population. The improved recognition and understanding of lucidity may enhance clinician education, caregiver guidance, and ethical decision-making in palliative and hospice care settings.

## Introduction and background

Since the 18th century, lucidity in the end stages of an individual’s life has been documented by several physicians and researchers. Historically, these accounts have drawn associations with brain abscesses and tumors, strokes, dementias including Alzheimer’s disease, meningitis, schizophrenia, and possible affective disorders [1]. These cases cover a wide range of ages, from a five-year-old boy with a malignant brain tumor to a 91-year-old woman who had experienced two strokes. Despite these findings, the general literature on lucidity and its subtypes has been fairly limited, with less than 70 case reports and general statements made by healthcare workers (e.g., physicians and caregivers) in existence prior to 1975. Yet, based on these reports, the vast majority of those experiencing lucid episodes (LEs), 41 out of 49 cases or 84%, had done so in their final day to week of life [2]. Although research on lucidity has been sparse prior to the 21st century, there has been an increased and unforeseen interest in this topic in recent years, prompting the creation of new research methods and definitions to refine our knowledge on lucidity.

Lucidity and its subtypes (i.e., paradoxical lucidity {PL} and terminal lucidity {TL}) throughout the literature have many different definitions. For example, Suzuki et al. describe it as “a temporary improvement in coma or disturbance of consciousness (due to organ failure) in a terminal ill patient lasting longer than 24 hours, with the patient achieving consciousness, lucidity, and the ability to convey messages such as gratitude or farewell to their family” [3]. The patients in these instances are able to communicate and express their thoughts clearly to their family members moments before their death. Another paper described it as a moment when a terminally ill patient has a sudden onset of energy, alertness, and mental clarity and regains meaningful memory function just before their death [4]. While these two definitions might be similar in the most basic form, they are inherently different. The 2022 article offered a more clinical definition emphasizing the underlying condition, duration, and specific functional abilities regained, such as the ability to communicate clearly [3]. Contrast that with the interpretation found in the 2023 paper, which is nonspecific as it does not provide a specific disease state or process, duration, and/or specific cognitive abilities regained [4].

Another definition of TL given by Perera is the phenomenon when an individual who is near the end of life experiences moments of mental clarity and regains the ability to communicate clearly and meaningfully [5]. Unlike the other definitions provided, Perera applied this definition to individuals with mental impairment and dementia [5]. The version of lucidity provided by Kim et al. comes in the form of a lucid experience (LE) [6]. LE is described as “an unexpected and meaningful personal interaction with a family member, a friend, or even a neighbor, when said person has lost the ability to do so.” Similarly, Teresi et al. used the term “paradoxical lucidity (PL)” to describe this phenomenon [7]. PL is described as a spontaneous ability to regain mental clarity, a clear form of communication, and a higher form of communication for a short period of time in a person who has lost the ability to do so. In distinguishing between paradoxical and terminal lucidity, the main differentiator is the proximity of the lucid episode to the patient’s death. However, it has also been asserted that a lucid episode must meet three criteria to fit the definition of paradoxical lucidity: “(1) a neurological condition that is assumed (2) to irreversibly (3) impair verbal or behavior interactions” [8]. It does not seem to matter if TL, PL, or LE is used; the basic inherent definition is the same, individuals with cognitive impairment briefly return to a level of normal mental functioning before succumbing to death, with slight differences in the specificities and the criteria required to meet the definitions proposed in each study (Table 1).

**Table 1:** Complete list of definitions of lucidity across selected articles* *Only 11 out of 19 articles chosen for this review have provided definitions for lucidity **The number of individuals who observed LEs or the total number of LEs reported according to the original authors’ definitions -Review articles; these do not report original data LEs: lucid episodes

Author(s) and Year	Definition	Number of LEs**
Normann et al., 2006 [9]	“Patients unexpectedly say or act in a way that surprises the care provider because the patients seem to be much more aware of their situation and to function much more adequately than usual”	52
Brayne et al., 2008 [10]	Sudden burst of energy	7
Claxton-Oldfield and Dunnett, 2018 [11]	“A patient who was unconscious, confused, or suffering from dementia becoming suddenly lucid enough to coherently say goodbye to their loved ones at the bedside”	26
Lim et al., 2020 [12]	Episodes in which there was a “reemergence of alertness from dull or unconsciousness states”	6
Peterson et al., 2022 [8]	“(1) A neurological condition that is assumed (2) to irreversibly (3) impair verbal or behavior interactions”	-
Suzuki et al., 2022 [3]	“A temporary improvement in coma or disturbance of consciousness (due to organ failure) in a terminal ill patient lasting longer than 24 hours, with the patient achieving consciousness, lucidity, and the ability to convey messages such as gratitude or farewell to their family”	32
Ijaopo et al., 2023 [4]	“A period when a critically ill person transiently experiences increased alertness, energy surge, and an unexpected return of mental clarity and memory functioning shortly before death”	-
Teresi et al., 2023 [7]	“Unexpected episodes of spontaneous mental clarity, such as the ability to communicate, in persons who had seemingly lost such abilities. This could include return to a higher level of communication, even if for a brief period”	29
Ramirez et al., 2024 [13]	“Brief periods of resurfacing ‘normal’ or heightened mental abilities/mental clarity and alertness; displaying augmented awareness of self and the contextual environment; and the ability of action, expression, and communication, like those present prior to the onset of cognitive decline”	13
Kim et al., 2025 [6]	“An unexpected, spontaneous, meaningful, and relevant communication with your relative, friend, or neighbor when they had lost the ability to speak or have personal interactions”	412
Perera, 2026 [5]	“A phenomenon where individuals near death, particularly those with cognitive impairment or dementia, experience sudden and unexpected moments of mental clarity and awareness”; “a temporary resurgence of cognitive functions that were previously believed to be lost”; “unexpected episodes of spontaneous, meaningful, and relevant communication or behavior in dementia patients who appear to have lost cognitive capacity”	-

Although the amount of literature on lucidity is currently quite limited, there have been proposed exploratory mechanisms for how lucidity and its terminal variant function. Among these come from Nahm, who provided two models: “(1) All cases of TL can be explained entirely by brain physiological processes. (2) Not all cases of TL can be explained entirely by brain physiological processes” [14]. As the neurobiological mechanisms behind lucidity have still not been adequately studied, there is currently no way to confirm which of the two, if any, stand true. However, there are several other proposals for how researchers should approach studying and characterizing lucidity, which include clinimetrics, psychometrics, and the Delphi method [15]. In the context of lucidity, clinimetrics would use clinical signs and symptoms and vital signs to create a standardized index for assessing lucidity, psychometrics would use standardized measurements and statistical analyses to establish important criteria for this phenomenon, and the Delphi method utilizes a more systematic approach to studying topics with limited data and experimental methods (e.g., literature review) by involving a panel of experts to provide their perspectives on the topic of interest. The current study aims to review recent studies using the above methods to compare the perspectives of family members to healthcare workers involved in the care of patients who have experienced LEs regarding their observations of these events.

## Review

Methods

**Figure 1:**
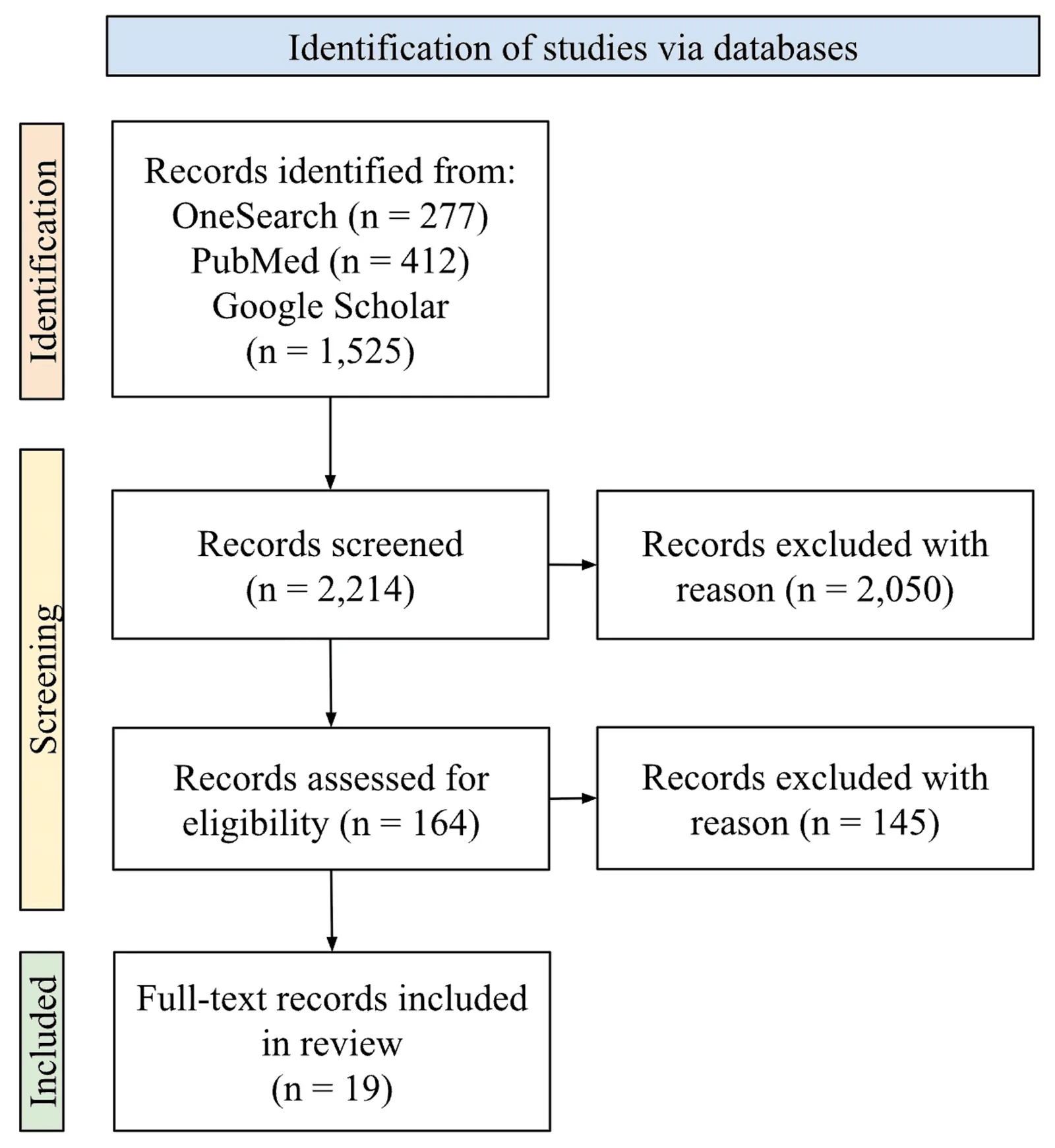
PRISMA flow diagram for selected articles

Through the use of several databases (e.g., PubMed and Google Scholar), primary and secondary sources were located using the following search terms: “terminal lucidity,” “paradoxical lucidity,” “lucidity,” “doctor,” “physician,” “family,” “family member,” “caregiver,” “healthcare,” and “healthcare worker.” These terms were used in combination with each other using the Boolean operators “AND” and “OR” (i.e., lucidity “AND” physician, terminal lucidity “AND” family member, and paradoxical lucidity “AND” family “OR” doctor). Due to the limited nature of the available literature, searches were expanded to include publication dates for full-text records spanning over the course of the last 30 years (n = 19). Despite this finite number of selected works, this is merely a reflection of the evolving nature of the approaches taken to evaluate LEs and should not be viewed as a drawback. Reasons for exclusion included research focused on delirium, cognitive decline, or a disease process without the implication of lucidity; nonempirical publications; and language and accessibility limitations. A detailed breakdown of how the articles were selected can be observed in Figure 1.

Results

General Observations

Observers of LEs in recent years fall under one of three categories: general population (e.g., no clear distinction whether observations came from family members or members of the healthcare team), family members, and non-family members (e.g., healthcare workers and professional caregivers). Regardless of observer type, patients experiencing LEs most commonly had advanced dementia or another severe neurological disorder, although similar episodes have also been described in pediatric oncology patients. Prior to these events, patients typically exhibited moderate to severe cognitive impairment, ranging from profound deficits in memory and communication to states of unresponsiveness or unconsciousness. Despite these baseline impairments, LEs consistently involved a transient return of awareness and purposeful communication lasting from several minutes to hours before patients returned to their prior condition or subsequently died.

One study that looked at these features was conducted by Batthyany and Greyson, who reported that patients had prominent cognitive impairments prior to the episode yet experienced transient improvements in communication lasting from minutes to days [16]. Similarly, Ramirez et al. found that both family and professional caregivers shared that patients had “enhanced verbal communication followed by the brief, unexpected quality of lucidity” accompanied by sadness, confusion, and surprise [13]. Although most studies focused on dementia, Roehrs et al. demonstrated that LEs may also occur in pediatric oncology patients, suggesting that lucidity is not limited to neurodegenerative disorders. In all three cases, children briefly regained expressive communication, emotional awareness, and an apparent understanding of their impending death following prolonged neurological decline [17].

The following sections provide further details regarding the similarities and differences between family members and non-family caregivers in relation to lucidity, specifically pertaining to the number of observed episodes and their durations, exhibited behaviors, potential triggers and events, and their responses to such episodes. Unless stated otherwise, all episodes of lucidity occurred in populations with Alzheimer’s disease or other forms of dementia. A summary of the most pertinent findings can be found in Table 2.

**Table 2:** Comparison of family members’ and healthcare workers’ pertinent observations of lucid episodes LEs, lucid episodes; TBI, traumatic brain injury

Observation	Family Members	Healthcare Workers
Characteristics	Meaningful yet temporary improvements in verbal communication, the recognition of relatives, awareness, behavior, emotional expression, and overall responsiveness [6,7,10,12,17-19]	
Duration and timing	Less than one minute to several days; most commonly occurred hours to days before death, with patients returning to baseline cognitive state [6,7,10-12,17,18]	
Frequency of reports	Substantially more LEs, likely due to longer periods spent with patients and broader episode interpretation [3,6,13,17,18]	Fewer LEs; generally applied stricter clinical criteria when identifying episodes [3,7,13]
Prior patient condition	Moderate to severe cognitive impairment, often with profound communication deficits [6,13,17,18]	Advanced neurological disease with impaired consciousness or cognition (e.g., dementia, Parkinson disease, stroke, and TBI) [7,10-12]
Perceived triggers	Visits from family or friends or other environmental factors, although many reported no identifiable trigger [6,18]	Spontaneous events without an identifiable trigger, often occurring during close caregiver-patient interactions [10,19]
Interpretation	Emotional responses, including hope, confusion, surprise, and the appreciation of remaining time; spouses more likely to change care decisions [4,6,13]	Clinical observations while attempting to separate emotions from professional interpretations [13]

Observations From Family Members

Characteristics of lucid episodes: Family members of those who experienced lucidity included spouses and adult children, taking on the role as primary caregivers for their relatives. Taking various studies into account, these family members had observed 490 lucid episodes based on their respective definitions [3,6,13,17,18]. Most lucid episodes that were observed by family members came from a 2025 study conducted by Kim et al., with 412 total episodes; 193 caregivers observed two episodes, and 26 individuals reported one [6]. Despite differences in study designs and patient populations, family caregivers usually described LEs as transient improvements in communication, awareness, and purposeful behavior occurring after periods of moderate to severe cognitive impairment.

Across these studies, patients commonly regained the ability to communicate effectively, recognize loved ones, demonstrate facial expressions, express emotions, and pursue purposeful goals. Karlawish et al. described verbal communication, the increased awareness of surroundings, the recognition of specific people and animals, the improved understanding of others’ emotions and intentions, and goal-directed behavior as common characteristics of LEs [18]. Similarly, Kim et al. reported that patients typically exhibited awareness with communication that made some or complete sense despite moderate to extreme cognitive impairment before the event [6]. Comparable findings were also observed in pediatric oncology patients described by Roehrs et al., in which all three children experienced sudden improvements in awareness and communication despite prolonged neurological decline. During these episodes, the children had substantial conversations with their parents, recognized their impending death, and provided their loved ones with reassurance and farewell messages before dying shortly afterward [17].

Although the duration of LEs varied between studies, most instances were brief. Kim et al. found that episodes generally lasted less than 10-30 minutes among family members [6], whereas Karlawish et al. reported episodes lasting from a few seconds to 45 minutes [18]. Following the episode, patients either returned rapidly to their prior level of cognitive impairment or died soon after; however, some survived for weeks or months following the event [6,17,18].

Potential triggers and clinical context: While family caregivers routinely attempted to identify precipitating factors for LEs, there were inconsistencies in what was reported to trigger these events. Kim et al. found that adult children were more likely than spouses to report episodes occurring after visits from family or friends [6]. Likewise, Karlawish et al. noted that most caregivers did not report an identifiable trigger prior to the LE, but some believed that such occurrences were linked to social interactions, medication changes, sleep quality, exercise, the time of day, or spontaneous brain activity [18]. Overall, these observations suggest that although caregivers often searched for an explanation, LEs appeared to occur unpredictably.

In these studies, family caregivers generally reported that patients displayed moderate to severe cognitive impairment before the episode, followed by a transient return of meaningful communication before rapidly returning to baseline [6,13,17,18]. These findings remained consistent despite differences in patient populations and episode duration.

Emotional interpretation of lucid episodes: Family caregivers commonly interpreted LEs within the emotional context of their loved one’s illness. Ramirez et al. found that caregivers initially attempted to define lucidity through an intellectual perspective, expressing curiosity regarding its cause while simultaneously describing sadness, confusion, and surprise [13]. When asked to describe lucidity, many caregivers emphasized the contrast between the expected disease progression and the sudden return of purposeful communication, which can be seen as an attempt to reconcile the unexpected nature of these events with the established understanding of the disease process [13].

Although family members had similar observations, there have been slight differences in reports between adult children and spouses of the patients. Kim et al. reported that spouses were more likely than adult children to describe nonverbal communication, view episodes in a negative manner, and alter care decisions following an LE, whereas adult children more commonly reported episodes lasting between 31 and 60 minutes and associated them with visits from family or friends [6]. Ramirez et al. also reported that caregivers often perceived LEs as providing a false sense of hope for recovery as patients returned to their previous level of impairment within one day or less [13]. However, Ijaopo et al. suggested that these episodes may provide families with opportunities to appreciate the time remaining with their loved ones while acknowledging the inevitability of death [4].

In general, family caregivers typically described LEs as brief episodes characterized by meaningful communication and awareness following advanced cognitive impairment. Although potential triggers remained inconsistent, caregivers typically held an emotional interpretation of these events, viewing them as unexpected deviations from the anticipated disease course while creating a sense of hope and confusion, as well as opportunities for profound final interactions [4,6,13,17,18].

Observations From Healthcare Workers and Non-family Caregivers

Recognition and frequency of lucid episodes: Compared with family members, healthcare workers generally reported fewer LEs and often applied stricter criteria when identifying them. Suzuki et al. found that among healthcare workers and professional caregivers, only one event out of 443 total events met their criteria for an LE [3]. The authors attributed this discrepancy to differences in observer exposure and the use of a separate questionnaire for healthcare workers and family members (i.e., healthcare workers were given the East Asian Collaborative Cross-cultural Study to Elucidate the Dying Process {EASED} questionnaire, whereas family members were given the Japan Hospice and Palliative Care Evaluation Study {J-HOPE4} questionnaire). Similarly, Ramirez et al. reported that only six out of 20 professional caregivers described episodes meeting the conceptual definition of lucidity despite 15 of these individuals providing examples of unusual patient behavior [13]. These findings suggest that healthcare workers may underrecognize LEs relative to family caregivers.

Clinical characteristics of lucid episodes: Despite differences in reporting frequency, healthcare workers described similar characteristics of LEs. Teresi et al. reported that 29 LEs were observed by physicians, nurses, certified nursing assistants, social workers, and physical therapists, with most patients experiencing cognitive impairments from a neurological disease process (i.e., Alzheimer’s disease, dementia, Parkinson disease, stroke, or traumatic brain injury {TBI}) [7]. During the episodes, patients had short-term improvements in expressive speech, receptive communication, awareness, and overall functioning despite advanced neurological disease. The lengths of the episodes ranged from less than a minute to several days, with most occurring in the morning or afternoon. Many patients either died shortly afterward or remained alive for up to three months following the episode [7].

Similar observations were reported by Lim et al., who identified only six LEs among 151 hospitalized patients. The authors noted that the patients were stuporous, drowsy, or comatose before the episode, followed by cognitive improvements lasting from several hours to four days before death [12]. Additionally, hospice workers surveyed by Claxton-Oldfield and Dunnet reported that LEs most commonly occurred within the final 24-48 hours of life [11]. Brayne et al. also found that nursing home staff observed LEs hours to days before death, with few episodes occurring weeks earlier [10]. Despite the varied durations of LEs, the healthcare workers who participated in these studies consistently observed LEs in patients with advanced neurological disease anywhere from minutes to days before their death.

Clinical interpretation and observed behaviors: Unlike family members, healthcare workers generally approached LEs from a clinical rather than emotional perspective. Ramirez et al. found that professional caregivers emphasized patients’ unexpected return of mental clarity while attempting to separate their personal emotions from their observations [13]. Although surprise remained a common reaction, staff members generally described LEs using clinical descriptors rather than personal interpretations.

In addition to the attempts to separate their emotions from their observations, healthcare workers also observed common behavioral features accompanying LEs. Brayne et al. reported that healthcare workers differentiated LEs from medication-induced hallucinations by noting purposeful communication, body language, and a sense of peace or comfort in affected patients [10]. Likewise, Normann et al. observed that most LEs occurred spontaneously during close interactions between a caregiver and patient, often within important environmental contexts (i.e., when the patient and caregiver worked closely together, in response to something happening in the patient’s surroundings) [19]. Reported behaviors included humor, gratitude, curiosity, happiness, longing, fear, and purposeful verbal communication [19]. In a subsequent population-based study, Normann et al. found that patients who experienced LEs showed greater emotional expression and behavioral engagement than those who did not experience these episodes [9].

Collectively, healthcare workers frequently described LEs as uncommon, spontaneous events characterized by temporary improvements in communication, awareness, and behavior among patients with advanced neurological disease. Compared to family caregivers, healthcare professionals generally emphasized the clinical features of these episodes and applied stricter criteria when identifying them, yet both groups reported similar behavioral characteristics once an LE was recognized [3,7,9-13,19].

## Conclusions

This study reviewed the current literature on LEs to identify common characteristics and potential predictors and compare observations made by family members and healthcare professionals. Individuals experiencing LEs typically have advanced neurological disorders with significant cognitive impairment, with a sudden, temporary return of cognitive function, including meaningful communication, increased awareness, and purposeful behavior. The defining feature of an LE is its brief nature, often occurring minutes to hours before death, though some are reported weeks to months earlier. While family members and healthcare professionals describe similar core features, they differ in the frequency and interpretation of the events. Potential triggers remain poorly understood and inconsistent across studies. Overall, LEs appear to be a genuine and recurring phenomenon that warrants further investigation to better understand their causes and implications for patient care.

Although lucidity has been described for centuries, the current evidence remains limited by definitional variations, retrospective reporting, small sample sizes, and the absence of physiological and neurobiological measures, reflecting the early stages of research in this field rather than methodological shortcomings. Future research should prioritize the development of standardized definitions, validated assessment tools, and prospective study designs to improve comparisons across studies and better characterize the timing, mechanisms, and clinical significance of LEs. Incorporating physiological and neurological monitoring during these episodes may further clarify their biological basis, including the possibility of transient alterations in neurotransmitter activity and neural network function. The improved recognition and understanding of LEs may enhance clinical education, guide communication with families, and support more informed ethical and end-of-life decision-making.
